# Impairments of Brain and Behavior: The Neurological Effects of Alcohol

**Published:** 1997

**Authors:** Marlene Oscar-Berman, Barbara Shagrin, Denise L. Evert, Charles Epstein

**Affiliations:** Marlene Oscar-Berman, Ph.D., is a professor in the departments of psychiatry and neurology, Boston University School of Medicine, and a research scientist in the Psychology Research Service, Department of Veterans Affairs Medical Center, Boston, Massachusetts. Barbara Shagrin, Ph.D., is a clinical assistant professor of psychiatry, Boston University School of Medicine, Boston, Massachusetts. Denise L. Evert, Ph.D., is a research associate in the department of psychiatry, Harvard Medical School, Boston, Massachusetts. Charles Epstein, Ph.D., is associate professor of neurology and director of clinical neurophysiology, Emory University School of Medicine, Atlanta, Georgia

**Keywords:** chronic AODE (alcohol and other drug effects), AODR (alcohol and other drug related) disorder, brain function, brain damage, nervous system disorder, brain imaging, neuropsychological assessment, cognitive process, emotion, memory, learning, personality, neurotransmitters, patient family history, malnutrition, liver disorder, aging, gender differences, drug therapy, hypothesis testing, literature review

## Abstract

Chronic heavy drinking and alcoholism can have serious repercussions for the functioning of the entire nervous system, particularly the brain. These effects include changes in emotions and personality as well as impaired perception, learning, and memory. Neuropathological and imaging techniques have provided evidence of physical brain abnormalities in alcoholics, such as atrophy of nerve cells and brain shrinkage. At the cellular level, alcohol appears to directly affect brain function in a variety of ways, primarily by interfering with the action of glutamate, gamma-aminobutyric acid, and other neurotransmitters. Neurological disorders also can result from vitamin deficiency and liver disease, two health problems that commonly occur with alcoholism. Other hypotheses, based on factors such as aging, gender, and genetics, have been developed to explain various alcohol-related neurological consequences. Many pharmacological treatments to improve neuropsychological functioning in alcoholics have been tested, but none has proved entirely successful. With prolonged abstinence, however, slow recovery of cognitive functioning can occur in some cases.

Alcohol consumption can damage the nervous system, including the brain. Consequently, alcoholics^1^ and chronic heavy drinkers can suffer abnormalities in their mental functioning and changes in behaviors associated with brain impairment. The neurological effects of alcohol can occur directly, because alcohol is a toxic substance, or they can occur indirectly, through damage to other body organs (e.g., the liver) that subsequently interferes with the workings of nerve cells in the brain (see figure 1).

Images of the brain created with modern neuroradiological techniques, such as magnetic resonance imaging (MRI) and computed tomography (CT), generally show a relationship between prolonged alcohol consumption and changes in the brain’s structure (Charness 1993; Pfefferbaum et al. 1995). For example, MRI and CT images have shown brain shrinkage and tissue damage (i.e., brain lesions) in some alcoholics. These changes can cause poor temperature regulation, muscle weakness, and alterations in sleep patterns.

This article reviews some of the physical brain changes and neuropsychological^2^ consequences of alcoholism, beginning with the effects of chronic alcoholism on memory and other cognitive functions. This discussion is followed by an examination of the differences among people that commonly contribute to the many neurological effects of alcoholism, including medical health, age, gender, and family history of alcoholism. The article concludes with a consideration of treatment and recovery.

## Common Effects of Alcohol on the Nervous System

Alcohol has effects on both major components of the nervous system—the central nervous system (i.e., the brain and the spinal cord) and the peripheral nervous system (i.e., the nerves in the rest of the body).

Alcohol can have a negative effect on certain neurological processes, such as temperature regulation, sleep, and coordination. For example, moderate amounts of alcohol lower body temperature. Severe intoxication in a cold environment may produce massive, life-threatening declines in temperature (i.e., hypothermia). Many people mistakenly believe that alcohol can help warm them in cold weather. This notion can be especially dangerous for the homeless, for elderly people living in inadequately heated quarters, and for those exposed to prolonged cold temperatures outdoors.

In addition to its effect on body temperature, alcohol interferes with normal sleep patterns. Relatively small doses of alcohol can cause early sedation or sleepiness, awaking during the night, and suppression of rapid-eye-movement (REM) sleep. REM sleep is the dreaming stage of sleep; when REM sleep occurs near wakefulness, it often produces vivid hallucinations. Most people fall asleep easily after one or more alcoholic drinks^3^ but experience diminution of REM sleep. Drinkers who attempt to use alcohol as a sedative seldom attain a full night’s sleep, however; after several hours, the natural elimination of alcohol from the body produces arousal and sleep fragmentation. When chronic drinkers withdraw from alcohol, long-suppressed REM sleep may rebound excessively. Some authorities (Greenberg and Pearlman 1967) believe that delirium tremens (known as DT’s), a condition occurring 2 to 4 days after alcohol withdrawal that consists of trembling and agitation with hallucinations, overexcitation, fever, sweating, and rapid heartbeat, represents a state of continuous REM sleep. In addition, measurable insomnia may occur many weeks into abstinence.

**Figure 2:**
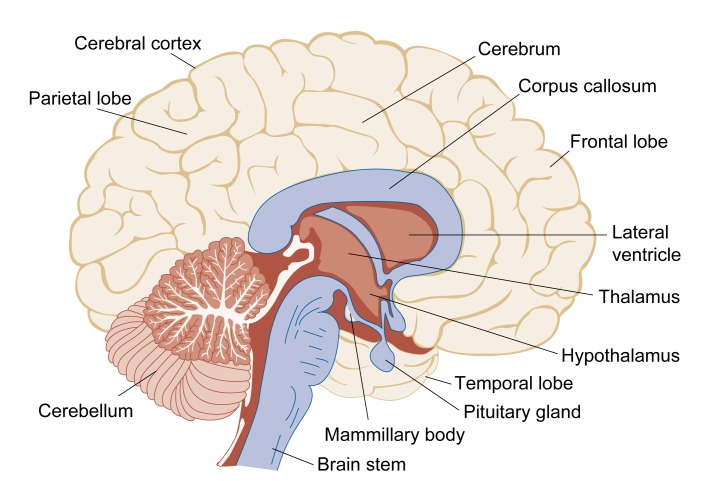
Schematic of a lengthwise cross-section through the human brain. Brain structures that most frequently have been implicated in alcohol-related neurological disorders include parts of the diencephalon (i.e., the mammillary bodies of the hypothalamus and the dorsomedial nucleus within the thalamus), the cerebral cortex, and several central neuro-transmitter (i.e.,nerve cell communication) systems.

Another prominent effect of chronic alcohol consumption is harm to the part of the brain called the cerebellum (figure 2), resulting mainly in the loss of muscular coordination. This damage appears as imbalance and staggering, although other problems also may occur (Raymond et al. 1996).

A peripheral nervous system disorder commonly seen in alcoholics is numbness and weakness in the hands and feet (i.e., peripheral neuropathy). This condition is thought to be largely a consequence of malnutrition in severe alcoholics. One type of peripheral nerve damage known as Saturday night palsy can occur when an alcoholic puts pressure on vulnerable nerves in the arm while lying in an intoxicated stupor, leaving him or her unable to extend the wrist for days to weeks.

## Abnormalities in Neuropsychological Functions

In addition to changes in temperature regulation, sleep, and coordination, alcoholism-related brain changes can cause abnormalities in mental functioning that are detectable using specialized neuropsychological tests. Behavioral neurologists and neuropsychologists use these sensitive tests to measure both the obvious and the subtle consequences of brain damage. Results of the tests often show changes in emotions and personality as well as impaired perception, learning, and memory (i.e., cognitive abilities) after damage to particular brain systems (see Evert and Oscar-Berman 1995).

### Korsakoff’s Syndrome

One of the most severe consequences of long-term alcoholism on mental functioning is Korsakoff’s syndrome (KS), a devastating memory disorder in which a person appears to forget the incidents of his or her daily life as soon as they occur (see Oscar-Berman 1990). Because of this dramatic loss of short-term memory (also called anterograde amnesia), patients with KS virtually live in the past. For example, someone who developed KS in the 1960’s might believe that the President of the United States is Dwight Eisenhower or John Kennedy. Some alcoholics may have a genetic component or predisposition to develop this amnesic condition: These patients may have an enzyme deficiency that prevents their bodies from using thiamine (a B vitamin) efficiently.^4^ This deficiency, coupled with a diet high in alcohol and low in thiamine (along with other nutrients), may lead to brain damage causing the amnesia.

Although KS destroys short-term memory, it typically spares most long-term memories (i.e., memories formed or knowledge gained before the onset of prolonged heavy drinking). Thus, overall intelligence, as measured by standardized IQ tests, does not necessarily deteriorate, because the types of information and abilities tapped by these tests usually involve long-term memory.

### Other Neuropsychological Problems

Within the past 25 years, clinical and experimental observations of patients with and without KS have revealed many other neuropsychological dysfunctions associated with alcoholism. Alcoholics demonstrate poor attention to what is going on around them; need extra time to process visual information; have difficulty with abstraction, problem-solving, and learning new materials; exhibit emotional abnormalities and disinhibitions; and show reduced visuospatial abilities (i.e., the capacity to deal with objects in two-dimensional or three-dimensional space) (Parsons and Nixon 1993). The once-common view that alcoholics without Korsakoff’s syndrome are cognitively intact has been abandoned in light of accumulating evidence that cognitive impairments (and associated changes in brain structure) can occur in alcoholics who do not exhibit obvious clinical signs of anterograde amnesia (see Lishman 1990).

## Alcoholism-Related Brain Damage and Associated Neuropsychological Changes

The type and extent of structural damage to brain tissue can be determined by autopsy (i.e., post mortem) examination of the brain’s components and individual nerve cells (i.e., neuropathological evidence). In addition, neuroradiological techniques, such as MRI and CT scans, allow the brain to be viewed inside the skull of a living person. Other neuroimaging techniques (i.e., functional neuroimaging) measure active brain functioning. Functional neuroimaging can reveal changes in the blood flow in and around the brain, brain metabolism, and brain electrical activity generated by nerve impulses (i.e., neurophysiological measures).^5^ One type of neurophysiological measure, event-related potentials (ERP’s), consists of brain waves recorded from scalp electrodes while a person is presented with specific pieces of information or stimuli. Scientists use computers to translate the information obtained from ERP’s and other functional neuroimaging measures into meaningful pictures that, in turn, make it possible to view brain functioning while a person is thinking or performing a task.

**Figure 3:**
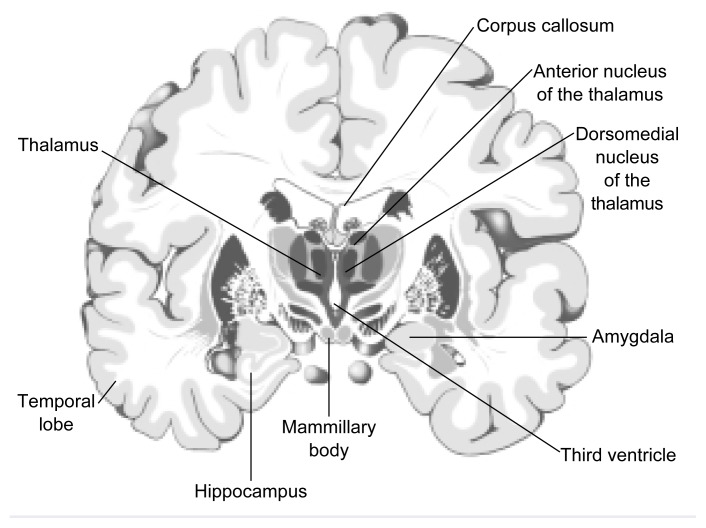
Schematic of a cross-section through the human brain. Brain structures that have been implicated in alcohol-related neurological disorders include parts of the limbic system (i.e., the hippocampus and the amygdala), the mammillary bodies of the hypothalamus, and the dorsomedial nucleus within the thalamus. SOURCE: Adapted from Nieuwenhuys, R.; Voogd, J.; and van Huijzen, C. *The Human Central Nervous System: A Synopsis and Atlas*. New York: Springer Verlag, 1988.

When applied to alcohol research, neuropathological and imaging techniques have helped to provide cumulative evidence of brain abnormalities in alcoholics, such as atrophy^6^ of nerve cells (i.e., neurons) and brain shrinkage (Hunt and Nixon 1993). Brain shrinkage appears as abnormal widening of the grooves (i.e., sulci) and fissures on the brain’s surface or enlargement of the fluid-filled cavities deep inside the brain (i.e., the ventricles). Regions of the brain that are especially vulnerable to damage after years of chronic alcoholism include the cerebellum, the limbic system (including the hippocampus and amygdala), the diencephalon (including the thalamus and hypothalamus), and the cerebral cortex (see figures 2 and 3).

Countless intricate pathways of neurons link the different areas of the brain, including the regions implicated in alcohol-related neurological dysfunction. Because of the size and complexity of this network, the consequences of damage to one structure or system often can resemble the consequences of damage to another. The following sections describe alcohol-related structural and neuropsychological changes that can occur in the brain.

### The Limbic System

The limbic system is an intricate network of structures located deep inside the brain; its functions are diverse and varied. One function of the limbic system receiving attention from alcohol researchers is memory. Memory loss similar to the amnesia in KS patients has been associated with damage to the hippocampus and the amygdala, parts of the limbic system that are located in the temporal lobes of the brain (see Evert and Oscar-Berman 1995). Although injury to the limbic system can cause amnesia, researchers are not certain of the degree to which alcohol-related memory impairments may be linked to damage in that part of the brain.

Alcohol researchers are interested in other functions of the limbic system as well. Damage to certain parts of the limbic system leads to abnormalities in emotional functioning, the sense of smell (i.e., olfaction), and the ability to use one sense (e.g., vision) to learn something in another sense (e.g., touch) (i.e., cross-modal functioning). In all these categories of function, researchers have observed deficits in alcoholics (Evert and Oscar-Berman 1995). Moreover, alcoholics with KS appear to have greater impairment in some of these functional areas than do non-KS alcoholics.

### The Diencephalon

The diencephalon, a region nestled in the center of the brain, acts like a way station for nerve signals moving from one area of the brain to another. Although it is not known precisely what role diencephalic structures play in human memory functioning, lesions in this region have been clearly documented in amnesic patients (Victor et al. 1989). Researchers are not certain whether alcohol-related memory impairments are caused by these lesions, however. An alternative explanation comes from a study that compared MRI measures of diencephalic damage in alcoholics with and without KS (Blansjaar et al. 1992). The authors suggested that diencephalic lesions develop regardless of whether patients acquire the amnesia of KS and are not so much typical of KS as they are of chronic alcoholism and malnutrition.

### The Cerebral Cortex

The cerebral cortex is the intricately folded outer layer of the brain composed of nerve cell bodies (i.e., gray matter). It is considered to be the center of higher consciousness and the seat of all intelligent behavior. The cortex makes neural connections, both directly and indirectly, with all parts of the nervous system and, therefore, with all parts of the body.

As noted previously, neuroradiological evidence has revealed a widening of the fissures and sulci of the cerebral cortex and enlargement of the ventricles in brains of alcoholics. These changes suggest cortical atrophy associated with alcoholism (Pfefferbaum and Rosenbloom 1993; Pfefferbaum et al. 1995). The evidence for cortical atrophy has come both from imaging studies of detoxified alcoholics and from post mortem analyses of the brains of alcoholics. For example, MRI findings show evidence of significant cortical and subcortical tissue and volume loss in non-KS alcoholics compared with nonalcoholic control subjects. Moreover, alcoholics with KS have greater cortical atrophy than non-KS alcoholics. Researchers also have reported neuropsychological deficits in alcoholics (e.g., through tests of problem-solving, spatial memory, visual associations, and learning related to or caused by touch [i.e., tactual learning]) that indicate alcoholism-related cortical atrophy (Evert and Oscar-Berman 1995).

In most studies of alcohol-related neurological disorders, researchers have assessed neuropsychological deficits in alcoholics without examining changes in alcoholics’ brains. To better understand brain-behavior relationships, however, neuropsychological, structural, and functional changes must be evaluated to relate changes in behavior to damage in particular systems of the brain. In studies using both methods, in fact, results have not revealed consistent relationships between cortical damage and performance on neuropsychological tests. Some measures of brain structure or function have correlated with cognitive test scores, whereas others have not. For example, one study reported a relationship between certain neuropsychological test scores and measures of frontal brain metabolism in long-term alcoholics; the same study, however, found no correlation between neuropsychological performance and degree of cortical atrophy as seen using MRI (Wang et al. 1993). The results were interpreted as reflecting either the preservation of cognitive abilities with mild structural brain changes or the insensitivity of the tests used to detect mild structural changes.

The most consistently and frequently reported findings in alcoholics, based on functional and structural imaging techniques, have been abnormalities in frontal brain regions (for reviews, see Oscar-Berman and Hutner 1993; Pfefferbaum and Rosenbloom 1993). Frontal-system functions include planning, carrying out, and monitoring goal-directed and socially suitable behaviors. Compared with nonalcoholic control subjects, some alcoholics have shown significant reductions in cerebral blood flow in certain areas of the frontal regions as well as in other brain areas. In addition, greater blood flow reduction in frontal cortical areas has been associated with greater severity of alcoholism and poorer cognitive test performance. In other studies, alcoholics showed diminished metabolic functions in frontal areas; this reduction was associated with impaired neuropsychological functions.

In studies of KS patients, researchers have obtained additional findings supporting frontal-system dysfunction (Oscar-Berman and Hutner 1993). Neuropsychological studies have shown that KS patients exhibit clinical signs associated with damage to the frontal cortex (e.g., emotional apathy, personality changes and loss of inhibitions, and constant repetition of certain responses despite feedback indicating that such responses are incorrect or inappropriate [i.e., abnormal response perseveration]). Although much debate centers on the connection between measures of alcohol consumption and the degree of structural or functional impairment in non-KS alcoholics, research so far has failed to demonstrate a clear connection between measures of alcohol intake, cognitive dysfunction, and frontal damage.

## Neurotransmitters and Alcoholism

At the cellular level, alcohol appears to affect brain function in a variety of ways. For example, alcohol can alter the action of the chemicals that allow neurons to communicate (i.e., neurotransmitters). Specialized proteins on the surface of neurons, known as receptors, recognize neurotransmitters and initiate the cell’s response. Neurotransmitters and receptors cluster where nerve cells come into close contact; these contacts are called synapses. Some neurotransmitters stimulate (i.e., excite) a response from the neurons that receive them; others inhibit neuronal response. Over periods of days and weeks, the levels of receptors change in response to chemical and environmental influences (e.g., drugs and synaptic activity) on the neurons. Genes in the neuron’s DNA are turned on or off, increasing or decreasing the synthesis of receptors. Over time, drugs that excite a given receptor generally lead to a reduction in (i.e., down-regulate) the numbers or activity of that receptor type. Drugs that inhibit a receptor eventually tend to lead to an increase in (i.e., up-regulate) that type of receptor. Up-and down-regulation are means by which the nervous system maintains a functional balance of neurotransmitters and receptors; when imbalances occur, effects can include seizures, sedation, depression, agitation, and other mood and behavioral disorders.

### Glutamate

The major excitatory neurotransmitter in the human brain is glutamate, an amino acid. Glutamate has a fundamental role in a cellular adaptation called long-term potentiation, which is a persistent increase in the efficiency of a neuron’s response to a neurotransmitter. Long-term potentiation may be an important mechanism in learning and memory.

Extremely small amounts of alcohol have been shown to interfere with glutamate action. This interference could affect multiple brain functions, including memory, and it may account for the short-lived condition referred to as “alcoholic blackout.” Because of its inhibitory effect on glutamate, chronic consumption of alcohol leads to up-regulation of glutamate receptor sites in the hippocampus, an area that is crucial to memory and often involved in epileptic seizures. During alcohol withdrawal, glutamate receptors that have adapted to the continual presence of alcohol may become overactive. Glutamate overactivity has been linked repeatedly to cell death in situations ranging from strokes to seizures. Deficiencies of thiamine and magnesium, which are common in alcoholics as a result of malnutrition, may contribute to this potentially destructive overactivity.

### GABA

Gamma-aminobutyric acid (GABA) is the major inhibitory neurotransmitter in the central nervous system. Evidence suggests that alcohol initially potentiates GABA effects; in other words, it increases inhibition, and often the brain becomes mildly sedated. But over time, chronic alcohol consumption reduces the number of GABA receptors through the process of down-regulation. When alcohol is eventually withdrawn, the loss of its inhibitory effects, combined with a deficiency of GABA receptors, may contribute to overexcitation throughout the brain. This effect, in turn, can contribute to withdrawal seizures (i.e., “rum fits”) within 1 or 2 days.

### Other Neurotransmitters

Alcohol directly stimulates release of the neurotransmitter serotonin as well as natural substances related to opioids (i.e., endorphins) that may contribute to the “high” of intoxication. Serotonin helps regulate functions such as food and water intake, sexual response, and aggression. Changes in other neurotransmitters, such as acetylcholine (which underlies key cardiovascular mechanisms, including dilation of blood vessels) and the catecholamines (the decreased transmission of which has been linked to the memory deficits of patients with KS [NIAAA 1993]), have been less consistently observed.

Alcohol disrupts neuron activity in various other ways. For example, over several weeks, alcohol reduces the level of nerve growth factors, proteins important for cellular adaptation and survival. In addition, alcohol may cause long-term adaptive changes in membrane lipids.

## Vulnerabilities to the Neurological Effects of Alcoholism

Alcoholism is a multidimensional disorder, and no simple answers exist to questions such as: “What are the neurological consequences of alcoholism?”; “What makes alcoholism affect different people in different ways?”; or even “What causes someone to become an alcoholic in the first place?” Widespread individual differences occur in the manifestation of alcoholism. For example, according to one estimate, 50 to 85 percent of non-KS alcoholics exhibit signs of cognitive decline (see Parsons 1993). Thus, anywhere from 15 to 50 percent of such alcoholics may not exhibit any obvious signs of cognitive impairment. In general, the greater the consumption of alcohol, the worse the performance on cognitive tasks. However, among those alcoholics who exhibit neurological problems, researchers have found that measures of previous alcohol consumption (e.g., duration, frequency, and quantity consumed) do not correlate consistently with the degree of neuropsychological dysfunction (Parsons 1993). This finding suggests that variables other than the presumed direct neurotoxic effects of alcohol may play a role in determining alcohol-related cognitive decline. In response to the variability in the consequences of alcoholism, researchers have looked for common elements that might help explain why certain alcoholics develop specific neurological symptoms or mental changes. Factors that may influence the neurological consequences of alcoholism include coexisting health problems, such as malnutrition and liver disease; the age at which problem drinking begins; the gender of the alcoholic; and a family history of alcoholism. These factors are considered in the sections that follow.

## Common Alcohol-Related Medical Problems

Two common health problems occurring with alcoholism are vitamin deficiency and liver disease, both of which can result in neurological disorders. As mentioned previously, prolonged drinking with improper diet and associated malnutrition can lead to thiamine deficiency, a possible factor in KS-related brain damage. Several investigators have stressed the idea that damage in the diencephalon of KS patients is caused by thiamine deficiency, whereas cortical abnormalities, most notably in the frontal lobes, are caused by alcohol neurotoxicity or other conditions frequently associated with alcoholism (e.g., liver disease or head trauma).

### Thiamine Deficiency

Researchers differ in their explanations of how and why particular neuropsychological deficits are displayed in alcoholics. One theory proposes that alcoholics may fall into subgroups distinguished by whether their brains are vulnerable to the direct neurotoxic effects of alcohol, to thiamine deficiency, or to both factors (Lishman 1990). According to this viewpoint, alcoholics who are susceptible to alcohol toxicity alone may develop permanent or transient cognitive deficits associated with cortical shrinkage. Those alcoholics who are susceptible to thiamine deficiency alone will develop a mild or short-lived KS state with anterograde amnesia as a salient feature. Alcoholics who suffer from a combination of alcohol neurotoxicity and thiamine deficiency (i.e., have dual vulnerability) will experience widespread damage to large regions of the cerebral cortex as well as to structures deep within the brain. These people will exhibit severe anterograde amnesia as well as other cognitive impairments.

### Liver Disease

Alcohol-related liver disease also contributes to neurological disturbances associated with heavy drinking (Tarter et al. 1993). The risk of alcoholic liver damage depends on factors such as the drinker’s nutrition, gender, and quantity and pattern of alcohol consumption. Recent research (Tarter et al. 1993) has focused on biological factors involved in protecting liver cells during metabolism; in some alcoholics, these protective mechanisms appear to be impaired. One condition associated with advanced liver disease, including alcoholic liver disease, is hepatic encephalopathy (also called portal-systemic encephalopathy [PSE]). PSE is a progressive metabolic liver disorder that affects intellectual functioning. Alcoholics with PSE have livers so damaged by cirrhosis that the flow of venous blood into the liver is obstructed, allowing toxic substances and metabolic by-products to enter the bloodstream. These toxins, which can include ammonia and manganese, circulate to the brain, where they interfere with the actions of neurotransmitters. The effects of PSE can be reversed to some extent with liver transplantation.

## Other Influences on Alcohol-Related Brain Injury

### Age

When researchers first began to study the effects of alcohol on the brain, they observed structural brain changes in alcoholics similar to those seen in nonalcoholic subjects as a result of normal chronological aging. These observations gave rise to the “premature aging hypothesis.” Two versions of the hypothesis exist, each with different propositions concerning the period in an alcoholic’s life during which premature aging begins (for reviews, see Ellis and Oscar-Berman 1989; Evert and Oscar-Berman 1995). According to the *accelerated aging* version of the hypothesis, aging starts to accelerate at whatever age problem drinking begins. This version predicts that young alcoholics will become old before their time and that neuropsychological and brain changes in alcoholics will mimic those found in chronologically older nonalcoholics. According to the *increased vulnerability* version of the premature aging hypothesis, vulnerability to alcohol-related brain damage is hastened only in people over age 50, in whom the normal manifestations of aging already have begun. This version suggests that because of the increased vulnerability of their brains to alcohol-related damage, older alcoholics will suffer more age-related symptoms and impairment than their nonalcoholic peers and younger alcoholics.

In the early observations from which the premature aging hypothesis evolved, researchers characterized the post mortem appearance of alcoholics’ brains as being small and shriveled compared with the brains of age-matched nonalcoholics (Courville 1966). The appearance was likened to the shrinkage that is associated with normal chronological aging. Other researchers, using neuroimaging techniques, have reported comparable findings in support of the accelerated aging hypothesis; backing the increased vulnerability hypothesis, older alcoholics displayed more brain tissue loss in brain scans than did younger alcoholics (see Pfefferbaum and Rosenbloom 1993). On the whole, most of the structural evidence supports a possible link between alcoholism and premature aging.

Unlike studies assessing brain atrophy, however, neuropsychological investigations have not accumulated much support for either version of the premature aging hypothesis. Results of a few studies favor the increased vulnerability hypothesis, but the evidence is inconsistent (for reviews, see Ellis and Oscar-Berman 1989; Evert and Oscar-Berman 1995). In support of the hypothesis, one study demonstrated that on the parts of IQ tests that normally pose difficulties for elderly nonalcoholic people, alcoholics between the ages of 48 and 74 performed significantly worse than same-age nonalcoholic control subjects and younger alcoholics (Ellis 1990). The increased vulnerability hypothesis leads to a second prediction, however, which was not supported by the study.

The second prediction hinges on the considerable evidence from a separate line of research into possible right-hemisphere brain dysfunction in alcoholics (see Oscar-Berman 1992). Like patients with damage to the right hemisphere, alcoholics typically perform poorly on visuospatial tasks. The similarity in performance between alcoholics and patients with right-hemisphere damage led researchers to hypothesize that right-brain functions are more vulnerable than left-brain functions to the effects of alcohol. Thus, the increased vulnerability hypothesis predicts that older alcoholics, the group in whom the effects of aging and alcoholism are combined, would show deficits out of proportion to their age on specialized tests of right-hemisphere functioning.^7^ This was not the case in the study just described (Ellis 1990). The results of numerous other studies examining right-hemisphere functional decline in relation to alcoholism and aging have not been sufficiently consistent to resolve the premature aging issue (for a review, see Ellis and Oscar-Berman 1989).

Regardless of alcohol’s role in aging, older alcoholics, by virtue of their chronological age, may be particularly susceptible to the effects of alcohol. For example, elderly alcoholics have an increased risk of accidents, deleterious side effects, and overt toxicity resulting from alcohol intake. Treatment for medical conditions common among the elderly (e.g., chronic pain and heart disease) also may increase alcohol-related problems in this group. For example, alcohol-medication interactions can have neuropsychological consequences ranging from drowsiness to disorientation; physical effects can include hemorrhage, malnutrition, and liver damage, which also can lead to neuropsychological problems.

### Gender

Controversy exists over whether and to what extent chronic alcoholism affects women’s brains differently from men’s brains (Glenn 1993). Results of studies using the same techniques to measure brain structure and function in men and women have been inconsistent. However, researchers have found evidence of similar degrees of brain shrinkage and impairment on tests of mental functioning in men and women, even though the women participating in the study had shorter drinking histories than the men (Lishman et al. 1987). Such evidence has led investigators to hypothesize that women’s brains may differ from men’s brains in their susceptibility to alcohol-related damage.

**Figure 4:**
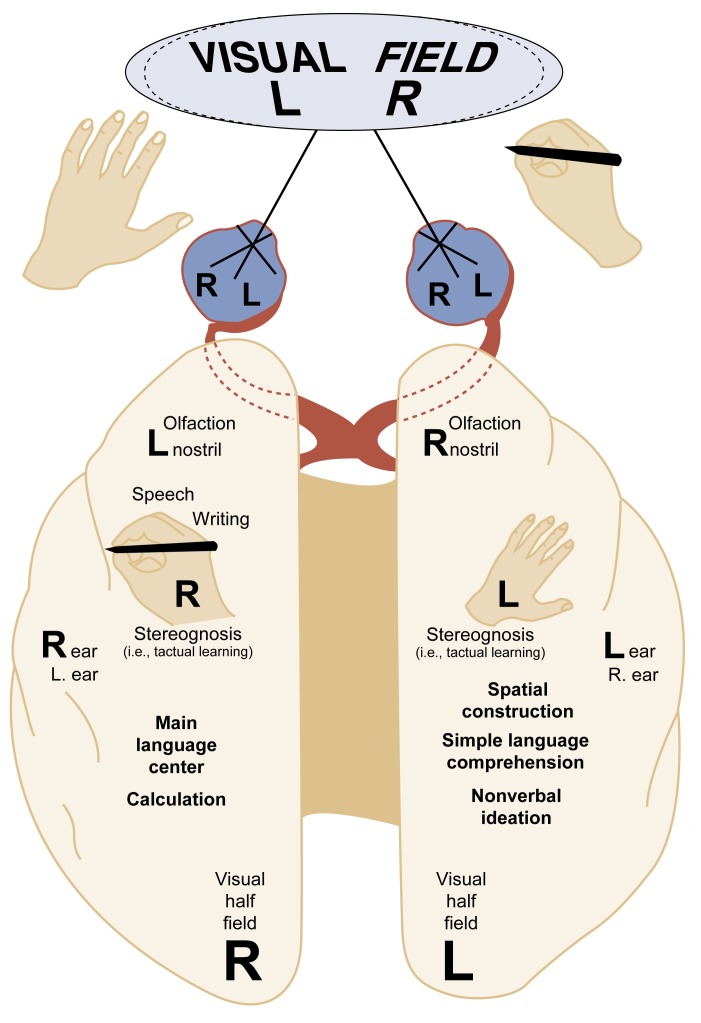
Schematic representation of perceptual and language functions in the right and left cerebral hemispheres of the human brain.

Since research suggests that alcohol may affect brain structure differently in men and women, one might also expect to see gender differences in the neuropsychological consequences of alcoholism. One way of studying possible neuropsychological disparities between male and female alcoholics is to examine gender differences in the functioning of the brain’s two hemispheres (i.e., differences in their functional cerebral laterality patterns). This question may be important because structural differences in men’s and women’s brains may be one factor underlying gender differences in perceptual asymmetries and other neuropsychological responses to alcohol. Normally, in both men and women, the left and right sides of the brain have disproportionate (i.e., asymmetrical) abilities to process linguistic (e.g., letters, words, and phrases) and non-verbal (e.g., visuospatial and musical) information. The left hemisphere usually is more efficient than the right with linguistic signals, and the right hemisphere is more efficient than the left for nonverbal signals. Scientists can study differences in hemispheric asymmetries using procedures called laterality tasks, which are sensitive to left and right hemisphere functioning. Figure 4 illustrates the neuroanatomy of human laterality (for a detailed description of laterality tasks, see Oscar-Berman 1992).

Laterality tasks allow researchers to conduct experiments in which conflicting visual, auditory, or tactual stimuli are sent simultaneously to the two halves of the brain. These tasks allow researchers to measure whether the left or the right side of the brain copes better with the competing information. With visual laterality tasks, the signals are presented on a computer screen; with auditory laterality tasks, the signals are presented through stereo earphones; and with touch tasks, the stimuli are given to the right and left hands. When research participants receive the stimuli, the side of the brain that is dominant for that material will favor the information coming into the side that is contralateral, or opposite, to that hemisphere. In experiments using auditory laterality tasks, for example, researchers may present two words (e.g., “bin” and “pin”) or two excerpts of music simultaneously to a subject, who then may be asked to identify the words or melodies he or she just heard. The left side of the brain, which is dominant for language, will favor words coming into the right ear, and the right half of the brain, which is dominant for music, will favor melodies coming into the left ear.

Studies comparing the separate functions of the left and right cerebral hemispheres have relied mainly on male research participants; no consistent pattern of abnormalities in alcoholics has emerged (for a review, see Oscar-Berman 1992). In a study that included both male and female alcoholics and nonalcoholic control participants, however, Drake and colleagues (1990) measured gender differences in hemispheric asymmetries using words and music presented simultaneously in each ear (i.e., dichotic listening). The investigators found that compared with control subjects, male alcoholics were better able to identify words coming into the right ear (a left-hemisphere function) and not as able to identify melodies coming into the left ear (a right-hemisphere function). In contrast, female alcoholics’ laterality patterns did not differ from those of control subjects on either of the dichotic listening tasks. The authors interpreted their results to mean that male alcoholics showed evidence of right hemisphere dysfunction. Results of numerous other perceptual laterality studies using visual, tactual, and auditory signals have been inconsistent in showing abnormal asymmetries in alcoholics; these studies, however, have not addressed gender differences (Oscar-Berman 1992). Continuing research on how alcoholism may affect the two halves of the brain differently in men and women may suggest gender-specific strategies for treatment.

### Family History

Researchers have found that adolescent and adult children of alcoholics who do not drink alcohol nevertheless show deficits in neuropsychological functioning (for reviews, see Babor et al. 1994; Porjesz and Begleiter 1993). Evidence suggests that children of alcoholics have difficulty regulating their own behavior, organizing and remembering information, and learning tasks that involve two- and three-dimensional space. In other studies, abnormal brain electrical activity, measured as a reduced peak in amplitude in one of the electrical components of the ERP (i.e., the P300 wave), has been observed in nondrinking sons of alcoholics who were performing cognitive tasks. Because the electrophysiological abnormalities in the children of alcoholics are similar to those displayed by abstinent alcoholics, researchers have inferred that brain waves may provide an observable marker for potential alcoholism in children of alcoholics even before the initiation of drinking behavior.

Family history of alcoholism has been associated with other notable results. In one study, intoxicated alcoholics, both with and without a family history of alcoholism, had problems on cognitive tests sensitive to temporal lobe functions (e.g., memory). On tests sensitive to frontal lobe functions (e.g., planning and judgment) however, only alcoholics with a positive family history of alcoholism performed poorly (Peterson et al. 1992).

## Recovery and Treatment

Studies suggest that slow recovery of cognitive functioning occurs in alcoholics who remain abstinent for at least 4 weeks, and certain indicators of impairment (i.e., CT and MRI images and brain glucose metabolism) have been shown to improve with prolonged abstinence (Pfefferbaum et al. 1995; Volkow et al. 1994). Although numerous pharmacological treatments have been given to alcoholics to improve neuropsychological functioning, none has proved entirely successful (Martin and Nimmerrichter 1993). Researchers have not established whether recovery is complete in most alcoholics (or what constitutes complete recovery), and they have not yet determined the typical length of the recovery period. With abstinence, some alcoholics show a slow reversal of neuropsychological impairment. Other alcoholics, however, display apparently irreversible deficits on specific tasks of cognitive function.

For example, in a study of cognitive recovery over a 14-month period, alcoholics who remained abstinent performed better than relapsers (Glenn et al. 1994), but abstainers did not perform as well as nonalcoholic control subjects. In another study, drinkers with a positive family history of alcoholism who had been abstinent for up to 4 months showed poorer performance on tests of cognition than either drinkers with a negative family history of alcoholism or abstainers with a positive family history (Drake et al. 1995). However, alcoholics both with and without a positive family history of alcoholism showed significant improvement with abstinence. Hence, a positive family history of alcoholism did not impede recovery of cognitive function among abstinent alcoholics. In most studies of neuropsychological deficits, length of abstinence typically has approximated 4 weeks. It is possible that past research may have overestimated permanent neuropsychological deficits related to chronic alcoholism by examining alcoholics whose mental functioning continued to improve following the studies’ conclusion.

## Summary and Conclusions

Several hypotheses have been proposed to explain the diversity of neuropsychological abnormalities shown by chronic alcoholics: (1) In patients with KS, alcoholism can selectively interfere with short-term memory, emotion, and other functions associated with damage to limbic system and diencephalic structures; and (2) alcoholics can also suffer diffuse cortical damage that affects the functioning of both brain hemispheres (e.g., abstracting and problem-solving abilities, poor attention, disinhibition, and perseverative responding). No definite relationships have been established, however, between damage to specific cortical regions and concurrent cognitive impairments, although findings from neuroimaging and neuropathology studies point to increased susceptibility of frontal brain systems.

Factors that contribute to differences among people in the neurological consequences of alcoholism are numerous and include nutritional deficiencies, liver disease, the age and gender of the drinker, and family history of alcoholism. The notion that neurological disorders result from the prolonged consumption of alcohol by certain vulnerable alcoholics is a plausible hypothesis, but identifying what makes certain alcoholics “vulnerable” remains a problem for further investigation.

Research on alcohol-related neurological disorders has centered on damage to the limbic system, diencephalon, and cerebral cortex. In addition, damage to central neurotransmitter systems has been considered as possibly contributing to alcohol-related abnormalities with harmful neurological consequences. Future research should help clarify the relative importance of the many biochemical effects of alcohol at all levels, from its effects on the preservation and replication of the genetic code embodied in DNA and the synthesis of new proteins, to the activities of neurotransmitters, receptors, neurons, and the entire brain. This information will link cellular changes directly to specific neurological consequences observed clinically. In the absence of a cure for alcohol addiction, a detailed understanding of the biochemical actions of alcohol on nerve cells may help in designing therapies to ameliorate its devastating neurological effects.

## Figures and Tables

**Figure 1:**
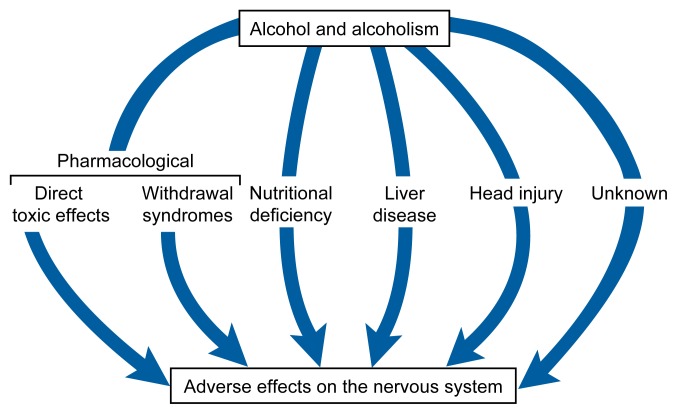
Sources of neurological complications of alcohol and alcoholism. SOURCE: Adapted from Bernat, J.L., and Victor, M. *The Neurological Complications of Alcohol and Alcoholism. Unit 7*. 2d ed. Developed by the Project Cork Institute at Dartmouth Medical School. Timonium, MD: Milner-Fenwick, 1994.

## Footnotes

1 The term “alcoholism” as used in this article refers to the criteria for alcohol dependence defined in the American Psychological Association’s *Diagnostic and Statistical Manual of Mental Disorders, Fourth Edition* (DSM–IV).

2 Neuropsychology is the branch of psychology focused on the study of the relationship between the brain and behavior.

3 One standard drink is defined as 12 fluid ounces (oz) of beer, 5 fluid oz of wine, or 1.5 fluid oz of distilled spirits.

4 Another disorder, Wernicke’s encephalopathy, frequently occurs with KS, leading to a diagnosis in the patient of Wernicke-Korsakoff syndrome. Patients with Wernicke’s encephalopathy exhibit confusion, uncoordinated gait, and abnormal eye movements, and imaging techniques reveal lesions in the diencephalon, the cerebellum, and the brain stem. Like KS, Wernicke’s encephalopathy is thought to be caused by a thiamine deficiency; the syndrome’s acute manifestations often can be reduced by thiamine administration. Hospitalized alcoholics being administered intravenous glucose also should receive thiamine to reduce the possibility of developing acute Wernicke’s encephalopathy.

5 For additional information on imaging techniques in alcohol research, see A*lcohol Health & Research World* Vol. 19, No. 4, 1995.

6 For definitions of this and other technical terms, see glossary, pp. 93–96.

7 Specialized tests that compare right-hemisphere functioning with left-hemisphere functioning are called laterality tests. Laterality tests are discussed in a later section; see also Oscar-Berman (1992).
